# COVID-19 in Somalia: Adherence to Preventive Measures and Evolution of the Disease Burden

**DOI:** 10.3390/pathogens9090735

**Published:** 2020-09-06

**Authors:** Mohammed A. M. Ahmed, Joseph Nelson Siewe Fodjo, Abdi A. Gele, Abdiqani A. Farah, Shariff Osman, Ibraahim Abdullahi Guled, Abdiaziz Mohamed Ali, Robert Colebunders

**Affiliations:** 1 Department of Paediatrics, Faculty of Medicine and Surgery, Mogadishu University, P.O. Box 004 Mogadishu, Somalia; ahmed.m@mu.edu.so (M.A.M.A.); shariff.osman@mu.edu.so (S.O.); drguledyare88@gmail.com (I.A.G.); shombe2001@yahoo.com (A.M.A.); 2 Department of Paediatric cardiology, Uganda Heart Institute, P.O. Box 7051 Yusufu Lule Road, Kampala, Uganda; 3 Global Health Institute, University of Antwerp, Doornstraat 331, 2610 Antwerp, Belgium; robert.colebunders@uantwerpen.be; 4 Department of Health Services, Norwegian Institute of Public Health, P.O. Box 222 Skøyen, 0213 Oslo, Norway; abdi.gele@fhi.no; 5 Puntland State University, Garowe, Somalia; abdiqani.farah@psu.edu.so; 6 Department of Paediatrics and Child Health, Mogadishu Somali-Turkey Training and Research Hospital, Mogadishu, Somalia; 7 De Martino Hospital, Mogadishu, Somalia

**Keywords:** COVID-19, Somalia, preventive measures, adherence, survey

## Abstract

Following the COVID-19 outbreak in Somalia, strict preventive measures were implemented by the government. We assessed adherence to the government recommendations via two consecutive online cross-sectional surveys between April and July 2020. A five-point adherence score was constructed based on self-reported observance of five preventive measures (physical distancing, face mask use, hand hygiene, mouth covering when coughing/sneezing, and avoidance of touching the face). 4124 and 4703 responses were analyzed during the first and second survey, respectively. The mean adherence score decreased from 3.54 ± 1.5 in the first survey to 3.40 ± 1.6 during the second survey; *p* < 0.001. More participants experienced at least one flu-like symptom during the second survey (38.2%) compared to the first (16.2%); however, the proportion of positive COVID-19 tests in the first (26.9%) and second survey (26.5%) was similar. The ordinal logistic regression model identified the following predictors for high adherence scores: female gender (odds ratio (OR) = 1.715 (1.581–1.861), *p* < 0.001); being a healthcare worker/student (OR = 2.180 (2.000–2.377), *p* < 0.001); obtaining COVID-19 information from official sources (OR = 1.460 (1.341–1.589), *p* < 0.001); and having postgraduate education (OR = 1.679 (1.220–2.307), *p* < 0.001). Conversely, obtaining COVID-19 information from social media and residing in urban settings were associated with lower adherence. Targeted and context-specific adaptations of the COVID-19 response may be required in Somalia.

## 1. Introduction

The novel coronavirus (COVID-19) disease outbreak has spread globally during the past months, posing significant public health challenges in virtually all countries. Resource-poor settings with weak healthcare systems, such as many sub-Saharan African (SSA) countries, find it difficult to adequately respond to the COVID-19 crisis, resulting in rapid human-to-human spread of the virus via respiratory droplets, contaminated objects, and direct physical contact with infected people [1]. Studies have shown that both asymptomatic and symptomatic infected persons can transmit the disease [2]. In the absence of effective treatments or vaccines, the World Health Organization (WHO) has strongly recommended that countries implement non-pharmaceutical interventions to attenuate the rapid spread of COVID-19 through minimizing contact between infected and uninfected persons. Suggested measures include lockdowns, closing schools and public places, physical distancing, the use of face masks, and stringent hand hygiene [3]. For the first time on a global scale, people are faced with travel restrictions, school closure, and the forbiddance of huge social gatherings. At the time of writing, the Americas and Europe bore about 70% of the global COVID-19 burden [4]. In Africa, the most affected country was South Africa (613,017 cases), followed by Nigeria (52,800 cases), and Ghana (43,717 cases); the least affected countries were the Seychelles, Eritrea, and Mauritius, with respectively 131, 315, and 346 reported cases as of August 26, 2020 [5].

Somalia is a SSA country that has a long history of internal conflicts, characterized by almost three decades of civil war. Currently, Somalia is among the countries of the world that are least able to cope with COVID-19 due to low levels of access to healthcare and limited government capacity. Healthcare services are either unaffordable, unavailable, or not trusted. At the onset of the pandemic, Somalia had no single laboratory with the capacity to diagnose coronavirus; hence, tests had to be transported to Kenya, with long waiting times before obtaining the results. Given the Ministry of Health’s (MOH) inadequate capacity in terms of qualified human resources, its preparedness for the pandemic was extremely poor. It was limited to temperature screening of people arriving through airports and isolating those suspected of having COVID-19. The first case was confirmed on March 16, 2020, followed by community transmission that was apparently initiated by first-line staff of the MOH, who were in contact with people arriving from abroad. Following the confirmation of the first COVID-19 case in Somalia, the government banned international and national flights, academic institutions were closed, and a public awareness campaign about the pandemic was initiated. Furthermore, the Somali government formed a COVID-19 task force, mainly for updating the public about the local evolution of the pandemic. Despite the implemented measures, the COVID-19 burden continued to rise in the country, with a cumulative 3269 cases and 93 deaths being officially reported by August 26, 2020 [4]. Considering that the government has focused its efforts on testing only suspected cases, the actual community burden of COVID-19 in Somalia has most likely been underestimated [6]. Of note, the country does not have a health research institute with the capacity to assess the development of the pandemic and guide the national response accordingly.

These unprecedented times found Somalia in a rebuilding stage, with most of the population living in heavily crowded and poorly constructed houses and large camps for internally displaced persons (IDP), with limited access to testing and/or healthcare [7]. The implementation of drastic COVID-19 preventive measures all over the national territory would likely infringe on the cultural and socio-economic norms of the people. Indeed, a large portion of Somalis rely on daily paid jobs for subsistence, and usually gather in mosques several times a day for prayers [7]. While initial reports suggest very poor compliance by the Somali people to the government’s instructions [8], it is currently unclear to what degree people will adhere to these measures over time, which factors determine adherence, and which specific interventions would rapidly contain the outbreak. Therefore, we conducted this online survey to assess how well the Somali residents respect the COVID-19 preventive strategies currently recommended by the government, as well as the possible impact on the disease burden of COVID-19.

## 2. Methods

### 2.1. Study Setting and Design

The study was carried out in Somalia (East Africa). Two consecutive cross-sectional surveys investigating the adherence to COVID-19 preventive measures were conducted. The first took place from 23 April to 7 May, whereas the second was held from 7 to 29 July 2020. The duration of the second survey was slightly longer because it took more time to recruit as many respondents as during the first survey. This study was part of a network of online surveys organized in several low and middle-income countries by the International Citizen Project on COVID-19 (ICPcovid) [9].

### 2.2. Survey Tool

The ICPcovid general online questionnaire was adapted to the Somali context by local investigators and was disseminated via social media platforms (in English and Somali) to consecutively recruit adult participants (age ≥ 18 years) via a snowball approach. All responses were submitted anonymously directly to the ICPcovid platform, mainly using smartphones but also via computers. Submitted responses were stored in the secure ICPcovid website until data extraction. In addition to socio-demographic information and self-reported adherence to various preventive measures, the questionnaire also included questions about the presence or absence of flu-like symptoms experienced by the respondents. Participants were asked whether they had been tested for COVID-19, and for the results of the test if available. A copy of the full questionnaire used is available as a Supplementary Material (File S1, Appendix 1).

### 2.3. Assessment of Adherence

Adherence to the government’s restrictive instructions for COVID-19 control were assessed using individual and community parameters, configured either as yes/no questions or as Likert scales in the questionnaire. A composite adherence score was made based on the respondent’s self-reported observance of the following personal preventive measures: physical distancing, face mask use, hand hygiene, coughing hygiene, and the habit of touching one’s face (Table 1).

### 2.4. Data Processing and Analysis

Descriptive statistics are presented using means with standard deviation (SD) for continuous outcomes, and percentages (%) for categorical variables. Pearson’s Chi-squared test was used to investigate associations between two categorical variables, and to compare proportions across the surveys. Continuous variables were assessed for normality using the Shapiro–Wilk test, and compared across groups using parametric tests (*t*-test, ANOVA) or non-parametric tests (Mann–Whitney U, Kruskal–Wallis) as appropriate. Both the WHO clinical definition (fever with at least one respiratory symptom) [10] and a recently proposed case definition (fever and/or anosmia, with at least one other respiratory symptom) [6] were used to estimate the COVID-19 burden in both surveys.

For multivariable analysis, we constructed an ordinal logistic regression model to investigate factors associated with adherence to national preventive measures against COVID-19, using the adherence score as the dependent variable. Mandatory covariates in the model included socio-demographic features (age, gender, profession, educational level, urban vs. rural residence), and survey timing (first vs. second survey). Non-Likert covariates which showed a significant association with the dependent variable in univariate analysis were also included in the model. The final model, selected based on the least Akaike information criterion (AIC) value, was obtained after a backward stepwise process (Supplementary File S1, Appendix 2). The significance level adopted was 5%, and all tests were two-sided. Data analysis was performed using the software R, version 3.6.2.

### 2.5. Ethical Considerations

The study protocol was approved by the University of Antwerp Ethics Committee (Ref: 20/13/148) and the Mogadishu University Institutional Review Board. Informed e-consent (checkbox) was required from each participant before submitting responses. All responses were anonymous and securely stored in a password-protected server in Belgium.

## 3. Results

### 3.1. Participant Characteristics

After data cleaning, over 4000 participants were retained in each survey round. Their comparative characteristics are summarized in Table 2. A majority of respondents during the second survey (2776/4703, 59.0%) acknowledged participating in the first survey as well. During both surveys, the study population was predominantly male, and most respondents had attained a university level of education. Based on data from the second survey only, respondents were geographically distributed across the regions of Somalia as follows: 3786 (80.5%) in Benadir; 227 (4.8%) in Galmudug; 212 (4.5%) in Hirshabelle; 168 (3.6%) in South West; 117 (2.5%) in Somaliland; 101 (2.1%) in Jubaland; and 92 (1.9%) in Puntland.

### 3.2. COVID-19 Preventive Behaviors

During the COVID-19 pandemic in Somalia, the participants reported low to moderate levels of worry/fear about their own health, with mean Likert scores on a five-point scale of 2.3 ± 1.6 and 1.9 ± 1.3 during the first and second survey, respectively (*p* < 0.001). A similar trend was observed regarding their level of worry/fear vis-à-vis the health of their loved ones (mean Likert scores on a five-point scale of 3.0 ± 1.7 during the first survey, and 2.2 ± 1.5 during the second survey; *p* < 0.001). Considering the individual COVID-19 preventive measures, the adherence rates ranged from 51.2% for mask use to 87.4% for coughing hygiene (Table 3). The mean adherence score decreased from 3.54 ± 1.5 in the first survey to 3.40 ± 1.6 during the second survey; *p* < 0.001. Combining available data from both surveys and comparing work habits in the private and public sectors during the COVID-19 pandemic in Somalia, we observed that 350/1041 (33.6%) of private employees and 91/176 (51.7%) of government employees were working from home (*p* < 0.001). Comparing COVID-19 preventive behaviours across genders revealed that women observed the safety recommendations more frequently than men (see Supplementary File S1, Appendix 3).

While some respondents reported using face masks all the time when they went out (29.9% in Survey 1 and 28.4% in Survey 2), others used masks only sometimes (19.4% in Survey 1 and 23.0% in Survey 2), and a minority wore masks at home (1.2% in Survey 1, 2.8% in Survey 2). About one quarter of the participants reported wearing reusable cloth (fabric) masks: 25.2% in the first survey, and 27.0% in the second survey. Among the 4071 participants who reported not using face masks during our surveys, the following reasons were given: no money to buy face masks in 933 cases (22.9%); not knowing where to get the face masks for 643 respondents (15.8%); the fact that face masks make them uncomfortable, as reported by 1096 (26.9%); whereas another 1695 (41.6%) thought that face masks are not necessary during the COVID-19 epidemic.

### 3.3. Difficulties to Observe the COVID-19 Lockdown Measures

The mean Likert score assessing the difficulty of observing lockdown measures increased from 2.2 ± 1.6 to 2.3 ± 1.5 in the first and second surveys, respectively (*p* < 0.001). In both surveys, the level of difficulty of staying at home, as required for lockdown purposes, was significantly different across genders, residential setting, and professional status; *p* < 0.001 (Table 4).

### 3.4. Flu-like Symptoms Reported by Participants

Overall, 667 (16.2%) participants in survey 1 and 1796 (38.2%) participants in survey 2 reported experiencing at least one flu-like symptom during the two weeks preceding the survey; *p* < 0.001. Headache was the most frequent symptom in both surveys (Table 5).

Applying the WHO’s clinical definition [10] and a recently proposed case definition [6] for COVID-19 screening (with no consideration of previous contacts with an infected person) revealed a prevalence of suspected COVID-19 cases ranging from 6.3%–8.1% in the first survey, and 6.5%–7.7% in the second survey (Table 6). During the second survey, mean adherence scores varied significantly across geographical regions (*p* < 0.001), ranging from 2.73 to 3.54.

### 3.5. Factors Associated with Adherence to COVID-19 Preventive Measures

A multivariable model investigating factors associated with the adherence score to COVID-19 preventive measures found that being a female, healthcare worker/student, and obtaining COVID-19 information from official sources significantly increased the odds for higher adherence (Table 7).

## 4. Discussion

This study shows an overall unsatisfactory level of adherence by Somali residents to the preventive measures put in place by the government to control COVID-19 transmission. Considering that more than half of the second survey participants also took part in the first survey, our findings may indeed reflect the evolution of COVID-19 preventive attitudes in a given cohort of individuals rather than in two different populations. The lower adherence scores during the second survey, compared to the first, indicates that compliance to government measures is decreasing as the COVID-19 epidemic evolves in Somalia. It appears that as the testing capacity increases, resulting in more COVID-19 cases being reported during the second survey, COVID-19 fear and adherence to preventive measures are slowly declining. The fact that the prevalence of suspected COVID-19 cases was fairly similar in the different regions of Somalia suggests that important community transmission of COVID-19 has been ongoing throughout the entire country [6]. This was to be expected, given the low adherence to preventive measures.

Our findings are particularly relevant for the population in the capital city Mogadishu, in the Benadir region, where over 80% of the respondents resided (data from Survey 2 only). Considering that Mogadishu is one of the few places in Somalia where the government healthcare system has actually deployed a COVID-19 response [8], it was not surprising to find relatively lower adherence scores in the other regions (except for Galmudug, which had slightly higher adherence scores than Benadir). Furthermore, the fact that urban residents and university level respondents were over-represented in both surveys makes it difficult to generalize the findings to the entire Somali population. It is estimated that less than 20% of Somalis aged 20 years and above have attained university level education [11]. This leaves a minority of the population fitting the characteristics of our survey participants. Notably, residing in rural areas was associated with more difficulties in complying with the government lockdown instructions, and with lower adherence scores. This may be due to the fact that rural residents are often involved in subsistence farming, and staying at home would imply dropping their main source of livelihood. It is therefore important to design targeted, context-specific COVID-19 prevention strategies that could be differentially implemented in different communities even within the same country.

The reported level of worry/fear about the respondents’ health or that of their loved ones significantly decreased from the first to the second survey. This was accompanied by reduced adherence scores across the surveys (Table 3). The initially high anxiety caused by the news of the high mortality of COVID-19 in developed countries with aging populations could be a possible explanation for this trend. However, upon realising that the disease is mostly benign among younger populations like in Somalia (barely 5% are older than 60 years [11]), the respondents may not be considering COVID-19 as a major threat anymore. Consequently, a progressive laissez-faire attitude was noted during the second survey, with more persons reporting that they have been frequenting public places such as bars/restaurants, places of worship, markets, or even travelled during the seven days prior to participating in the survey. The hypothesis of declining adherence as the epidemic evolves is further supported by the lower adherence scores observed among participants who have experienced flu-like symptoms, as it appears that they are no longer scared of the disease and hence are becoming less careful. Although younger persons may be at a lower risk for severe COVID-19, they can still become infected and transmit the disease to the high-risk groups (elderly and chronically sick individuals). Furthermore, COVID-19 deaths have been reported in persons below 40 years, although less frequently compared to older patients [12]. The WHO Director General recently highlighted that it is difficult to convince young people that they may indeed be at risk of dying from COVID-19, and urged them also to observe the preventive measures [13]. In a like manner, the youthful Somali population should be sensitized about the importance of adhering to preventive measures as much as possible for the sake of their own health and of the health of their loved ones.

About half of the respondents reported wearing face masks during the COVID-19 epidemic. This is far below the 80% threshold recommended by modelling studies [14], and therefore needs to be improved. Of note, reusable cloth masks were reported only in about one quarter of participants. Cloth masks offer some advantages, such as lower cost and being far less dangerous for the environment when compared to surgical masks, and have therefore been proposed for COVID-19 control especially in resource-poor settings [15]. Aside from the reported difficulties of acquiring masks (such as lack of finances and not knowing where to obtain masks), other major limitations to wearing masks included the associated discomfort and the widespread thought that masks were unnecessary in times of COVID-19. Indeed, masking is a relatively strange practice among the African population, and they should be educated about its importance during the COVID-19 crisis. This is in contrast with Asian countries, where masks are culturally accepted as a common hygienic practice [16].

The multivariable analysis showed several factors associated with adherence to COVID-19 preventive measures. The male gender was associated with poorer adherence, most likely because in the Somali community, men are often the breadwinners and are compelled to leave the house even in the midst of the COVID-19 outbreak, in order to work to support their families. This finding is in line with the results of a study in Spain, where it was also observed that women were more adherent to COVID-19 preventive measures than men, suggesting a more responsible attitude of women toward the dangers associated with the pandemic [17]. Respondents with a post-graduate educational level, who were probably more informed and knowledgeable about the disease, were more likely to have higher adherence scores compared to their less educated peers. In addition, government employees were more likely to highly adhere to the preventive measures compared to privately employed workers. We surmise that this is because the government agencies were more rigorous in implementing strategies against COVID-19 in the professional milieu (including working from home), compared to private companies, which are often out to maximize profit even at the detriment of employee welfare. Therefore, it is necessary that strict protocols that align with COVID-19 safety regulations be implemented and re-enforced in workplaces during the ongoing health crisis. It is equally worth mentioning that obtaining COVID-19 information from official sources (radio, TV, and government announcements) increased the odds of having a high adherence score, whereas information from social media decreased these odds. This highlights the role of misinformation in determining people’s adherence to scientifically proven preventive measures to control the COVID-19 epidemic [18]. Previous research has also demonstrated that social media users tend to be more extraverted [19], suggesting that such individuals may find it more difficult to stay at home and observe the COVID-19 preventive measures compared to the more introverted non-social media users.

## 5. Limitations of the Study

It is necessary that our findings be interpreted in the light of some limitations. First, the participants were not a representative sample of the general population in Somalia. Indeed, the majority of respondents were educated people belonging to the middle to high social classes and most often residing in urban settings, since poor people in remote places may have very limited internet access. Based on data from a recent demographic survey in Somalia [11], our study population constituted a minority (only 38.3% of Somalis are aged 18 years and above); our gender distribution was different (60% male in this study vs. 48.7% male in the general population); education levels were biased (>85% attended university in this study vs. 5.9% in the general population); and urban residents were over-represented (90% in this study vs. 63.9% in the whole country). Second, it is not possible to verify the veracity of responses obtained via a web-based questionnaire. Third, the fact that our study was done only at two time points makes it difficult to fully understand the rapidly changing landscape of the COVID-19 epidemic and related attitudes in Somalia. More studies, possibly with a longitudinal design, would be necessary to monitor the situation prospectively.

## 6. Conclusions

Our study revealed low adherence to COVID-19 preventive measures among residents of Somalia, with a decreasing trend in adherence over time. Mass sensitization and targeted interventions (based on the residential setting, profession, educational level, and gender) may be needed to improve preventive behaviours and limit the transmission of the virus. Additionally, social media platforms should be leveraged to disseminate accurate information about COVID-19.

## Figures and Tables

**Table 1:** Composition of the adherence score to COVID-19 preventive measures.

Variable	Scoring		Interpretation
I follow the 1.5–2m physical distance rule	Yes	1	1 point for yes, 0 point for no
	No	0	
I wear a face mask when I go out	Yes	1	1 point for yes, 0 point for no
	No	0	
I wash my hands regularly or I use hand sanitizer regularly	Yes	1	If any (or both) questions have answer yes, give just 1 point. Otherwise, give 0 point.
	No	0	
When I cough/sneeze, I cover my mouth and nose	Yes	1	1 point for yes, 0 point for no
	No	0	
I avoid touching my face (eyes, nose, mouth)	Yes	1	1 point for yes, 0 point for no
	No	0	
Total adherence score (maximum): 5			

**Table 2:** Participants’ characteristics during the two surveys in Somalia.

Characteristics	Survey 1 Participants *n* = 4124	Survey 2 Participants *n* = 4703	*p*-Value *
Age: Mean (SD)	22.9 (5.5)	23.7 (6.2)	<0.001
Gender: *n* (%)			
Male	2490 (60.5%)	2768 (59.1%)	0.188
Female	1626 (39.5%)	1916 (40.9%)	
Other/NA	8	19	
Education level: *n* (%)			<0.001
Primary school	20 (0.5%)	133 (2.8%)	
Secondary school	285 (6.9%)	562 (11.9%)	
University: Undergraduate	3429 (83.1%)	3506 (74.5%)	
University: Postgraduate	390 (9.5%)	502 (10.7%)	
Residential setting: *n* (%)			<0.001
Rural	47 (1.1%)	129 (2.7%)	
Sub-Urban	202 (4.9%)	371 (7.9%)	
Urban	3875 (94.0%)	4203 (89.4%)	
Live alone in household: *n* (%)	246 (6.0%)	282 (6.0%)	0.987
Profession: *n* (%)			<0.001
Student	2929 (71.0%)	3064 (65.1%)	
Unemployed	360 (8.7%)	598 (12.7%)	
Self-employed	197 (4.8%)	366 (7.8%)	
Private employee	563 (13.7%)	561 (11.9%)	
Government employee	75 (1.8%)	114 (2.4%)	
Working from home: *n* (%) ^a^	1527/2275 (67.1%)	1467/2938 (49.9%)	
Healthcare worker or student: *n* (%)	1089 (26.4%)	1589 (33.8%)	
Source of COVID-19 information: *n* (%) ^b^			
Radio, Television, or government	3042 (73.8%)	3225 (68.6%)	<0.001
Social Media	3264 (79.1%)	3045 (64.7%)	<0.001
Smoking: *n* (%)	97 (2.4%)	388 (8.3%)	<0.001
Underlying chronic disease: *n* (%) ^c^	246 (6.0%)	492 (10.5%)	<0.001
Tested for COVID-19: *n* (%)	208 (5.0%)	867 (18.4%)	<0.001
Tested during the past 2 weeks: *n* (%)	NA	215/867 (24.8%)	NA
COVID-19 Test results: *n* (%)			
Positive	49/182 (26.9%)	206/776 (26.5%)	0.992
Negative	133/182 (73.1%)	570/776 (73.5%)	
..Not known/NA	26	91	

*n*: number; NA: Not Applicable; * Chi squared test for categorical variables, Mann-Whitney U test for continuous variables. ^a^ Applicable only to workers; ^b^ Each participant was allowed to choose more than one answer, hence the categories may overlap; ^c^ Heart disease, diabetes, hypertension, cancer, HIV, or asthma.

**Table 3:** COVID-19 preventive behaviours.

	Survey 1 *n* = 4124	Survey 2 *n* = 4703	*p*-Value *
Wear face masks: *n* (%)	2112 (51.2%)	2644 (56.2%)	<0.001
Observe 1.5m physical distancing: *n* (%)	2634 (63.9%)	2781 (59.1%)	<0.001
Regular handwashing: *n* (%)	3334 (80.8%)	3491 (74.2%)	<0.001
Regular use of alcohol-based gel: *n* (%)	2352 (57.0%)	2687 (57.1%)	0.940
Cover mouth after coughing/sneezing: *n* (%)	3606 (87.4%)	3875 (82.4%)	<0.001
Avoid to touch face (eyes, nose, mouth): *n* (%)	2819 (68.4%)	2995 (63.7%)	<0.001
Staying home when I feel flu-like symptoms: *n* (%)	3464 (84.0%)	3418 (72.7%)	<0.001
Adherence score: *n* (%)			<0.001
0	168 (4.1%)	249 (5.3%)	
1	344 (8.3%)	514 (10.9%)	
2	484 (11.7%)	641 (13.6%)	
3	689 (16.7%)	720 (15.3%)	
4	983 (23.8%)	869 (18.5%)	
5	1456 (35.3%)	1710 (36.4%)	
Been to a bar/restaurant in the past 7 days: *n* (%)	525 (12.7%)	1837 (39.1%)	<0.001
Been to a market in the past 7 days: *n* (%)	2153 (52.2%)	2948 (62.7%)	<0.001
Been to a religious gathering in the past 7 days: *n* (%)	984 (23.9%)	1715 (36.5%)	<0.001
Travelled during the past 7 days: *n* (%)			<0.001
No	3907 (94.7%)	3876 (82.4%)	
Yes, to another province	155 (3.8%)	535 (11.4%)	
Yes, to another country	62 (1.5%)	292 (6.2%)	

* Chi squared test.

**Table 4:** Comparison of difficulty to stay at home across socio-demographic groups.

Variable	Categories	Survey 1		Survey 2	
		Mean Likert Score (SD)	*p*-Value *	Mean Likert Score (SD)	*p*-Value *
By gender	Male	2.39 (1.6)	<0.001	2.48 (1.5)	<0.001
	Female	1.84 (1.5)		2.46 (1.5)	
By residential setting	Rural	2.87 (1.8)	0.019	2.53 (1.4)	0.037
	Suburban	2.18 (1.6)		2.38 (1.5)	
	Urban	2.16 (1.6)		2.32 (1.5)	
By professional status	Student	2.03 (1.5)	<0.001	2.22 (1.5)	<0.001
	Unemployed	2.16 (1.6)		2.33 (1.5)	
	Self-employed	2.81 (1.7)		2.74 (1.5)	
	Private employee	2.62 (1.6)		2.60 (1.6)	
	Government employee	2.61 (1.6)		2.75 (1.5)	

* Mann Whitney U or Kruskal Wallis test as appropriate.

**Table 5:** Self-reported flu-like symptoms within the two weeks preceding each survey.

Symptoms: *n* (%)	Survey 1 *n* = 4124	Survey 2 *n* = 4703	*p*-Value *
Fever	360 (8.7%)	658 (14.0%)	<0.001
Headaches	442 (10.7%)	918 (19.5%)	<0.001
Dry cough	155 (3.8%)	230 (4.9%)	0.011
Productive cough	146 (3.5%)	194 (4.1%)	0.171
Sore throat	216 (5.2%)	356 (7.6%)	<0.001
Coryza	300 (7.3%)	397 (8.4%)	0.047
Loss of smell (anosmia)	278 (6.7%)	345 (7.3%)	0.295
Loss of taste (ageusia)	230 (5.6%)	282 (6.0%)	0.427
Shortness of breath	86 (2.1%)	150 (3.2%)	0.002
Myalgia	305 (7.4%)	345 (7.3%)	0.947
Fatigue	254 (6.2%)	251 (5.3%)	0.107
Nausea	119 (2.9%)	182 (3.9%)	0.013
Diarrhea	70 (1.7%)	126 (2.7%)	0.002

* Chi-squared test.

**Table 6:** Suspected COVID-19 prevalence based on clinical definitions and mean adherence scores by survey and by region.

Sub-Population: *n* (Mean Adherence Score)	Suspected COVID-19 Cases Based on WHO Definition [10]	Suspected COVID-19 Cases Based on Proposed Definition [6]	*p*-Value *
**Adherence score and suspected COVID-19 prevalence by survey round: *n* (%)**			
Survey 1: *n* = 4124 (3.54)	259 (6.3%)	334 (8.1%)	0.001
Survey 2: *n* = 4703 (3.40)	304 (6.5%)	361 (7.7%)	0.024
Differences across surveys *	0.758	0.486	
Adherence score and suspected COVID-19 prevalence by region: *n* (%) **			
Benadir: *n* = 3786 (3.43)	248 (6.6%)	296 (7.8%)	0.043
Galmudug: *n* = 227 (3.54)	12 (5.3%)	13 (5.7%)	0.852
Hirshabelle: *n* = 212 (3.41)	14 (6.6%)	16 (7.6%)	0.689
Jubaland: *n* = 101 (2.71)	6 (5.9%)	7 (6.9%)	0.772
Puntland: *n* = 92 (3.32)	7 (7.6%)	8 (8.7%)	0.786
Somaliland: *n* = 117 (3.03)	7 (6.0%)	8 (6.8%)	0.803
South West: *n* = 168 (3.07)	10 (6.0%)	13 (7.7%)	0.538
Differences across regions *	0.988	0.948	

* Chi-squared test; ** Data about regions available for the second survey only.

**Table 7:** Ordinal logistic regression model for Adherence score.

Covariate	Adjusted OR (95% CI)	*p*-Value
Age	0.994 (0.987–1.00)	0.126
Gender Male Female	Ref 1.715 (1.581–1.861)	<0.001
Profession Private employee Student Unemployed Self-employed Government employee	Ref 1.256 (1.109–1.423) 1.166 (0.994–1.369) 1.189 (0.989–1.430) 1.393 (1.058–1.839)	<0.001 0.059 0.066 0.019
Residential setting Rural Sub-Urban Urban	Ref 0.826 (0.602–1.131) 0.720 (0.541–0.956)	0.235 0.024
Educational level Primary Secondary Undergraduate Postgraduate	Ref 1.099 (0.800–1.510) 1.211 (0.898–1.632) 1.679 (1.220–2.307)	0.558 0.209 0.001
Healthcare student/worker	2.180 (2.000–2.377)	<0.001
Living alone in house	1.243 (1.056–1.463)	0.009
Presence of flu symptoms	0.678 (0.620–0.741)	<0.001
Presence of underlying disease	0.868 (0.751–1.004)	0.056
COVID-19 information from TV, radio, or government (Official source) No Yes	Ref 1.460 (1.341–1.589)	<0.001
COVID-19 information from social media No Yes	Ref 0.835 (0.764–0.913)	<0.001
Survey round Round 1 Round 2	Ref 0.898 (0.829–0.972)	0.008

OR: Odds ratio; CI: Confidence interval; Ref: Reference category.

## Supplementary Materials

The following are available online at https://www.mdpi.com/2076-0817/9/9/735/s1, Supplementary File 1: Appendix 1 (Questionnaire); Appendix 2 (Model AIC during stepwise process); Appendix 3 (Preventive behaviours by gender).
