# Supplementary material for: COVID-19 in Somalia: Adherence to Preventive Measures and Evolution of the Disease Burden
Source: Pathogens. 2020 Sep 6;9(9):735. doi: 10.3390/pathogens9090735 (PMC7560173; doi:10.3390/pathogens9090735)
Supplement: Supplementary file 1 [file pathogens-09-00735-s001.pdf]

SUPPLEMENTARY MATERIAL: COVID-19 ADHERENCE STUDY IN SOMALIA

APPENDIX 1: QUESTIONNAIRE

Socio-demographic information/ Macluumaadka Qofka

Q: Age/Da'da (*)

Type: number

A: number (min: 1 / max: 110 / step: 1)

Q: Sex/ Jinsiga (*)

Type: choice

A: one of the following:

male	=>	*Male/Lab*
female	=>	*Female/Dhedig*
other	=>	*Othe/Wax Kale*

Q: Nationality /Jinsiyadda (*)

Type: choice

A: one of the following:

local	=>	*I am a local/Waxaan ahay maxalli/muwaadin*
foreigner	=>	*I am a foreigner/Waxaan ahay ajnabi*

Q: Religion/Diinta (*)

Type: choice

A: one of the following:

christian	=>	*Christian/Masiixi*
muslim	=>	*Muslim/Muslim*
other	=>	*Other/Wax kale*
none	=>	*None/Midna*

Q: Highest educational level/Aqoonta waxbarasho (*)

Type: choice

A: one of the following:

primary	=>	*Primary/Dugsi hoose/dhexe*
Secondary	=>	*Secondary/Dugsi sare*

University Undergraduate degree holder	=>	*University Undergraduate degree holder/Jaamici shahaadada koowaad*
University Postgraduate degree holder	=>	*University Postgraduate degree holder/Jaamici shahaadada sare*

Q: Marital status/Xaaladda Guurka (*)

Type: choice

A: one of the following:

single	=>	*Single/Doob*
legally_married	=>	*Legally married/sharciyan xaasle*
cohabitation	=>	*Cohabitation/Qof la nool*
divorced	=>	*Divorced/Garoob*
widow_widower	=>	*Widow/widower/Carmali*

Q: Who do you currently live with? (many answers possible)Yaad hadda la nooshahay? (Jawaabo badan ayaad bixin kartaa) (*)

Type: choice_multiple

A: multiple answers possible:

My parent(s)	=>	*My parent(s)/Waalidiinteyda*
My spouse/partner	=>	*My spouse/partner/Xaaskeyga ama saaxiib*
My child(ren)	=>	*My child(ren)/Caruurteyda*
My sibling(s) or other relative(s)	=>	*My sibling(s) or other relative(s)/Walaalahey ama qaraabo kale*
Friends	=>	*Friends/saaxiibbadey*
I live alone	=>	*I live alone/Kalideey ayaan noolahay*

How many housemates do you have (yourself not included)? Please write the number of persons corresponding to each age group.

Write 0 if there is nobody in your home within a given age group.

Imisa qof ayaad la nooshahay (oo adigu aanad ku jirin)?Fadlan qor tirada dadka adoo ku saleynaya da'dooda qor 0 haduusan jirin da'da laxadiday

Q: Adults over 70 years of age: /Qof weyn ka weyn 70 sano:(*)

Type: number

A: number (min: n/a / max: 10 / step: n/a)

Q: Adults between 18 and 70 years: /Qof weyn u dhaxeeya 18 iyo 70 sano:(*)

Type: number

A: number (min: n/a / max: 10 / step: n/a)

Q: Children between 12 and 17 years:/Caruur u dhaxeysa 12 iyo 18 sano: (*)

Type: number

A: number (min: n/a / max: 10 / step: n/a)

Q: Children younger than 12 years: /Caruur ka yar 12 sano: (*)

Type: number

A: number (min: n/a / max: 10 / step: n/a)

Q: Do you live in:/Ma waxaad ku nooshahay: (*)

Type: choice

A: one of the following:

rural_area	=>	*a rural area/village/baadiye/tuulo*
suburb	=>	*suburb/Nawaaxiga magaalo*
provincial_town	=>	*provincial town/Degmo goboleed*
city	=>	*city/Magaalo*

Q: What are your housing conditions?/Waa sidee Xaaladdaada Deegaan? (*)

Type: choice

A: one of the following:

house_with_garden	=>	*a house or an apartment with a garden /Guri ama abaartiment/dabaq jardiino leh*
house_without_garden	=>	*a house or an apartment without a garden /Guri ama abaartiment/dabaq bilaa jardiino ah*
apartment_with_balcony	=>	*an apartment with a balcony / abaartiment/Dabaq barande leh*
apartment_without_balcony	=>	*an apartment without a balcony /Dabaq bilaa barande ah*
room	=>	*a room /Qol*
hut	=>	*a hut /Cariish, aqal laamayeeri iwm*
shack	=>	*a shack /Buul*
homeless	=>	*homeless /Guri ma degani*

Q: Which of the following can be found in your household? (multiple answers possible)/Waxyaabaha soo socda midkee ayaa laga heli karaa gurigaaga? (Jawaabo badan ayaad bixin kartaa) (*)

Type: choice_multiple

A: multiple answers possible:

bicycle	=>	*Bicycle/Baaskiil*
motorbike	=>	*Motorbike/Mooto*
car	=>	*Car/Baabuur*
radio	=>	*Radio/Raadiyo*
television	=>	*Television/ teleevishin*
phone	=>	*Mobile phone/ mobeel*
computer	=>	*Computer/ Kombuyuutar*
fridge	=>	*Fridge/ Firinjeer*

Daily life during the coronavirus epidemic /Nolol maalmeedka xilliga coronavirus ka

Q: Have you heard about the coronavirus epidemic? Mamaqashay safmareenka corona virus ? (*)

Type: choice

A: one of the following:

yes	=>	*Yes /Haa*
no	=>	*No /Maya*

Q: If yes, how did you hear about the coronavirus epidemic? (many answers possible)/ Hadday haa tahay, sideed ku maqashay cudurka faafa ee coronavirus? (Jawaabo badan ayaad bixin kartaa) (*)

Type: choice_multiple

A: multiple answers possible:

family_friends	=>	*From family, neighbours or friends/Familkayga/daris/saaxiib*
radio	=>	*From the radio /Raadiyaha*
television	=>	*From the television/ teleefishinka*
social_media	=>	*From the social media (WhatsApp, Facebook, Twitter, etc)/Baraha bulshada*
government	=>	*From government authorities/ Dowladda*
chief	=>	*From the village/quarter chief/Masuulka xaafadda*
religious_leader	=>	*From religious authorities / Sheekha*
health_personnel	=>	*From healthcare workers (nurses, doctors, etc) /Bahda caafimaadka*

Visible if

Q:	A:
Have you heard about the coronavirus epidemic? Mamaqashay safmareenka corona virus ?	- value => yes

Q: How many people apart from your housemates did you talk to yesterday face to face (not by phone, chat etc)?/Meeqa qof, oo aan kula nooleyn, ayaad toos ula hadashay shalay (kuma jiro telefoon, jaat iwm) (*)

Type: number

A: number (min: n/a / max: n/a / step: n/a)

Q: When was the last time you shook hands, gave a kiss or had any form of physical contact with someone other than a housemate?/Goormey ahayd markii ugu danbeysey ee aad gacanqaadday, shumisay/dhunkatay, amaba jirkiinnu is taabtay qof aan kula dagganeyn? (*)

Type: choice

A: one of the following:

today	=>	*Today/Maanta*
last_two_days	=>	*Last two days/Labadii maalmood ee ugu danbeysey*
last_3_to_6_days	=>	*Last 3 to 6 days/ 3 - 6 maalmood ee ugu danbeysey*
more_than_one_week	=>	*More than one week ago/ In ka badan hal toddobaad ka hor*
no_contacts	=>	*No contacts with persons outside my household/ Isma taaban qof ka baxsan dadka ila nool*

Q: During the last week did you have difficulties in obtaining food?/Toddobaadkii na dhaafay dhib ma kala kulantay sidaad cunto ku heli laheyd? (*)

Type: choice

A: one of the following:

yes	=>	*Yes /Haa*
no	=>	*No /Maya*

Q: What was the most important reason you had difficulties in obtaining food last week? (*)

Type: choice

A: one of the following:

no_money	=>	*Lack of money to go for shopping/Lacag la'aan*
little_food_available	=>	*Little food available in shops, market/Cunto-yari ka jirtay dukaamada, suuqa*
expensive	=>	*Food has become too expensive/ Cuntada ayaa qaali noqotay*
unsafe_to_go_out	=>	*I felt it was unsafe to go out to buy food /Waxaan dareemay in bedqaybkaygu anu ku jirin inaan u baxo si aan cunto u soo gato*
to_ill_to_go_out	=>	*I was too ill to go out /Aad ayaan u xanuunsanaa oo ma bixi karin*

Visible if

Q:	A:
During the last week did you have difficulties in obtaining food?/Toddobaadkii na dhaafay dhib ma kala kulantay sidaad cunto ku heli laheyd?	- value => yes

Q: During the last week, how worried or afraid were you about your health?:/Toddobaadkii na dhaafay, sideed uga walwalsaneyd ama ugu cabsaneysey caafimaadkaaga adoo ku cabbiraya 1= ma walwalin ilaa 5= aad ayaan u walwalay: (*)

Type: choice_scale

A: 1 = not worried/ma walwalin to 5 = extremely worried/ aad ayaan u walwalay

1	=>	*1*
2	=>	*2*
3	=>	*3*
4	=>	*4*
5	=>	*5*

Q: Have you suffered any form of violence or discrimination because of the measures taken against the coronavirus? (many answers possible)/Maxaa xadgudub ama faquuq ah oo aad ka cabanaysaa sababo la xiriira talaabooyinkii looga hortagayey Coronovirus ka? (*)

Type: choice_multiple

A: multiple answers possible:

home_violence	=>	*Physical violence at home/ Xaqgudub jirka oo guriga kula kulantay*
outside_violence	=>	*Physical violence outside/ Xaqgudub jirka oo aad banaanka kula kulantay*
socio_economic_discrimination	=>	*Discrimination because of my social/economic status/ Faquuq sababo la xiriira xaaladayda bulsho /dhaqaale*

ethnic_racial_discrimination	=>	*Discrimination because of my ethnicity, race or nationality/Faquuq sababo la xiriira isirkayga ama dhalashadayda*
no_violence_discrimination	=>	*No violence or discrimination/ Lama kulmin wax xadgudub ama faquuq ah*

Q: How did you arrange the care of your children today? /Sidee ayaad u agaasintay daryeelka caruurtaada Maanta?(*)

Type: choice

A: one of the following:

home_by_myself	=>	*At home, by myself/ Guriga, anaa agaasimay*
home_housemates	=>	*At home, with my housemates/Guriga, Dadka guriga igula nool ayaa ila agaasimay*
school_childcare	=>	*To school / childcare/ Iskuulka/ Daryeelka Caruurta*
friends_acquaintances	=>	*At friends / acquaintances/ Saaxiibo/ Macrifo*
aunts_uncles	=>	*At aunts or uncles/ Eedooyinkay/Habaryaraday ama Adeeraday/abtiyaday*
grandparents	=>	*At grandparents/Ayeeyday iyo Awoowgay*
home_house_help	=>	*At home, with a house help/nanny/ Shaqaale guriga*
other	=>	*Other/ Wax kale*
not_applicable	=>	*Not Applicable (no children at home)/ Caruur guriga ilama joogto*

Professional life during the coronavirus epidemic/ Nolashaadii shaqda intii uu jiray safmarka coronavirus

Q: What do you do for a living?/Maxaad ka shaqayn jirtay? (*)

Type: choice

A: one of the following:

student	=>	*Student/Arday*
jobless	=>	*Jobless/Shaqo la'aan*
self_employed	=>	*Self-employed/Anaa iskay u shaqeysan jiray*
company	=>	*Work for a person, institution or company/Waxaan u shaqeyn jiray qof, hay`ad ama shirkad*
government	=>	*Work for the government/ Dowladda ayaan u shaqeyn jiray*

Q: Are you a healthcare worker or a student working in the health sector?/Miyaad tahay shaqaale caafimaad ama arday ka shaqeynaya qeyb caafimaad? (*)

Type: choice

A: one of the following:

yes	=>	*Yes /Haa*
no	=>	*No /Maya*

Q: What are your current working conditions?/Waa sidee Hadda Xaaladahaaga Shaqo? (*)

Type: choice

A: one of the following:

worker_from_home	=>	*Worker from home/Shaqaale Guriga ka soo shaqeeya*
worker_open_space	=>	*Worker in an open space (market, shop, roadside, etc)/Shaqaale ka shaqeeya goob furan (suuq, dukaan, laamiga dhiniica, iwm)*

worker_closed_space_alone	=>	*Worker in a closed indoor space alone (office, etc.)/Shaqaale ka shaqeeya keligiis goob xiran (xafiis ,iwm)*
worker_closed_space_people	=>	*Worker in a closed indoor space with several people (office, etc.)Shaqaale kala shaqeeya dad kale goob xiran (xafiis,iwm)*
not_applicable	=>	*Not applicable (if jobless or student)/ ima khusayso (qof ah shaqo la'aan ama arday)*

Q: How many days per week do you usually go to school or work?/Meeqa maalin ayaad badanaa toddobaadkii tagtaa iskuulka ama shaqada? (*)

Type: number

A: number (min: n/a / max: 7 / step: n/a)

Q: How many days did you (physically) go to school or work last week?/Meeqa maalin ayaad tagtay iskuulka ama shaqadda toddobaadkii la soo dhaafay? (*)

Type: number

A: number (min: n/a / max: 7 / step: n/a)

Q: Are you working from home today?/Maanta ma guriga ayaad shaqadaadii ku qabanaysaa? (*)

Type: choice

A: one of the following:

yes	=>	*Yes /Haa*
no	=>	*No /Maya*
not_applicable	=>	*Not applicable (if jobless or student)/ima khusayso (qof ah shaqo la'aan ama arday)*

Q: Why are you not working from home?/maxaad shaqada guriga ugu dhex qaban wayday? (*)

Type: choice

A: one of the following:

not_possible	=>	*It is not possible with my job /Shaqadeydu kuma habboona*
not_allowed	=>	*It is possible, but is not allowed by my employer/Wey ku habboon tahay laakiin madaxdeydu ma oggola*
leave_house_make_money	=>	*I have to leave the house to make money to support my family/Waa in aan guriga ka baxo si aan lacag u soo shaqaysto oo aan qoyskeyga ku biilo*
home_not_working	=>	*I am at home but not working/ Guriga ayaan joogaa laakiin ma shaqaynayo*
no_risk_to_go_out	=>	*I don't think there is a risk to go out/U malayn maayo in khatar loo bixi waayaa ay jirto*
other	=>	*Other/wax kale*

Visible if

Q:	A:
Are you working from home today?/Maanta ma guriga ayaad shaqadaadii ku qabanaysaa?	- value => no

Q: What transportation means did you use to go to work?/Waa maxay nooca gaadiidka aad ku shaqo tagto? (*)

Type: choice

A: one of the following:

public_transport	=>	*By public transport with multiple people (train, bus, taxi, etc)/Gaadiidka dadweynaha oo dad badan raacan (tareen. Bas, taksi, iwm.)*
hired_vehicle	=>	*Hired a vehicle for myself and/or family members (private taxi, rented car, etc)/Gaari ayaan ijaartay ama u ijaaray qoyskayga(tagsi, gaari kiro ah, iwm.)*
own_transport	=>	*Own transport (vehicle, motorcycle, bicycle)/Gaadiid aan leeyahay (gaari, mooto, baaskiil)*
walked	=>	*Walked to work/Shaqada waan u lugeeyay*

Visible if

Q:	A:
Are you working from home today?/Maanta ma guriga ayaad shaqadaadii ku qabanaysaa?	- value => no

Q: During the last week, how much revenue loss did you suffer because of the coronavirus epidemic and related measures?/Toddobaadkii la soo dhaafay, immisa khasaara dakhliga ah ayaa ku soo gaaray oy sabab utahay iyo tallaabooyinka la xiriira safmarka coronavirus? (*)

Type: choice_scale

A: On a scale of: 0 = no losses; to 10 = 100% losses/ 0=khasaara la'aan, 10=100% khasaara ah

1	=>	*1*
2	=>	*2*
3	=>	*3*
4	=>	*4*
5	=>	*5*
6	=>	*6*
7	=>	*7*
8	=>	*8*
9	=>	*9*
10	=>	*10*

Personal preventive measures for coronavirus/Tallaabooyinka ka-hortagga shaqsiyeed ee coronavirus

Q: I wear a face mask when going outside/Waxaan xirtaa maaskiga wajiga marka aan dibadda u baxayo(*)

Type: choice

A: one of the following:

yes	=>	*Yes /Haa*
no	=>	*No /Maya*

Q: If yes, which kind of mask do you use? /Hadday haa tahay, maaska noocee ah ayaad isticmaashaa?(*)

Type: choice

A: one of the following:

disposable	=>	*Disposable mask/Maaskaro la tuuri karo*
reusable_cloth	=>	*Reusable cloth mask/Maaskaro dib loo isticmaali karo*
professional	=>	*Professional (strong, specialized) mask/Maaski Xirfadle (adag, khaas ah)*

Visible if

Q:	A:
I wear a face mask when going outside/Waxaan xirtaa maaskiga wajiga marka aan dibadda u baxayo	- value => yes

Q: If yes, when/where do you wear face masks?/Hadday haa tahay, goorma / xagee ayaad urxirtaa waji xidhka wejiga? (*)

Type: choice_multiple

A: multiple answers possible:

sometimes	=>	*Sometimes when I go out/Mararka qaar markaan baxo*
all_the_time	=>	*Every time I go out/Markasta oo aan baxo*
home	=>	*At home/Guriga*
work	=>	*At work/shaqada*

Visible if

Q:	A:
I wear a face mask when going outside/Waxaan xirtaa maaskiga wajiga marka aan dibadda u baxayo	- value => yes

Q: If no, why don't you use face masks?/Hadday maya tahay, maxaad u adeegsan weji xidhka? (*)

Type: choice_multiple

A: multiple answers possible:

money	=>	*I don't have money to buy face masks/Ma haysto lacag aan ku gato maaskiga wajiga*
where_to_get	=>	*I don't know where to get a face mask/Ma aqaan meesha laga helo maaskiga wejiga*
uncomfortable	=>	*Face masks make me uncomfortable*
unnecessary	=>	*I don't think that face masks are necessary/Uma maleynayo in maaskiga wajiga loo baahan yahay*

Visible if

Q:	A:
I wear a face mask when going outside/Waxaan xirtaa maaskiga wajiga marka aan dibadda u baxayo	- value => no

Q: I follow the social 1.5-2m meters distance rule/Waxaan raacaa qaanuunka kalafogaanshada bulshada 1.5-2 mitirka ah (*)

Type: choice

A: one of the following:

yes	=>	*Yes /Haa*
no	=>	*No /Maya*

Q: When I cough or sneeze, I cover my mouth and nose with a tissue paper/Marka aan qufaco ama hindhiso, waxaan afka iyo sanka ku daboolaa tiish iwm (*)

Type: choice

A: one of the following:

yes	=>	*Yes /Haa*
no	=>	*No*

Q: When I cough or sneeze, I usually wash/desinfect my hands immediately afterwards/Markaan qufaco ama hindhiso, waxaan markiiba dhaqaa ama jeermisdilaa gacmaheyga (*)

Type: choice

A: one of the following:

yes	=>	*Yes /Haa*
no	=>	*No /Maya*

Q: I measure my body temperature at least twice a week/Waxaan cabbiraa heerkulka jirkeyga ugu yaraan laba goor toddobaadkii (*)

Type: choice

A: one of the following:

yes	=>	*Yes /Haa*
no	=>	*No /Maya*

Q: I wash my hands using soap and water regularly during the day/ waxaan ku dhaqaa gacmaheyga biyo iyo saabuun si joogto ah maalintii (*)

Type: choice

A: one of the following:

yes	=>	*Yes /Haa*
no	=>	*No /Maya*

Q: I use a hand sanitizer regularly during the day/Waxaan isticmaalaa jeermisdilha gacmaha si joogto ah maalintii (*)

Type: choice

A: one of the following:

yes	=>	*Yes /Haa*
no	=>	*No /Maya*

Q: I avoid touching my face (eyes, nose and mouth)/Waxaan ka digtoonaadaa in aan taabto wajigeyga, (indhaha, sanka iyo afka) (*)

Type: choice

A: one of the following:

yes	=>	*Yes /Haa*
no	=>	*No /Maya*

Q: I disinfect my phone whenever I return home/Waxaan jeermisdilaa telefoonkeyga mar kasta oo aan guriga ku soo noqdo (*)

Type: choice

A: one of the following:

yes	=>	*Yes /Haa*
no	=>	*No /Maya*

Q: I stay home when I feel flu-like symptoms/Gurigaan joogaa markaan dareemo wax hargab u eg(*)

Type: choice

A: one of the following:

yes	=>	*Yes /Haa*
no	=>	*No /Maya*

Q: How difficult is it for you personally to follow the protective measure of staying home as much as possible?/Sidey kuugu adag tahay shaqsi ahaan, in aad guriga joogtid si aad uga hortagtid fiditaanka cudurka intii suurta gal ah? Adoo ku cabbiraya 1= dhib iguma aha 5= aad ayey iigu adag tahay (*)

Type: choice_scale

A: 1 = not difficult at all <=> 5 = extremely difficult

1	=>	*1*
2	=>	*2*
3	=>	*3*
4	=>	*4*
5	=>	*5*

Community preventive measures for coronavirus/Tallaabooyinka looga hortagayo coronavirus ee bulshada

Q: Approximately how many times did you wash your hands or use a hand sanitizer yesterday?/Qiyaastii meeqa goor ayaad gacmahaaga dhaqday ama isticmaashay jeermisdile shalay?: (*)

Type: number

A: number (min: n/a / max: 100 / step: n/a)

Q: Were you in a meeting or gathering with more than 10 persons during the last 7 days?/Ma ka qaybgashay shir ama kulan in ka badan 10 qof toddobaadkii la soo dhaafay? (*)

Type: choice

A: one of the following:

yes	=>	*Yes /Haa*
no	=>	*No /Maya*

Q: Did you go to a restaurant, bar, club, dancing, party, or concert during the last 7 days?/Ma aadday makhaayad, baar, kalaab, cayaar, xaflad, ama riwaayad toddobaadkii ugu danbeeyey? (*)

Type: choice

A: one of the following:

yes	=>	*Yes /Haa*
no	=>	*No /Maya*

Q: Did you go to a religious gathering during the last 7 days?/Ma aadday kulan diimeed toddobaadkii ugu danbeeyey?(*)

Type: choice

A: one of the following:

yes	=>	*Yes /Haa*
no	=>	*No /Maya*

Q: Did you attend a funeral the last 7 days?/Ma ka qaybgashay duug/aasitaan qof dhintay toddobaadkii la soo dhaafay?(*)

Type: choice

A: one of the following:

yes	=>	*Yes /Haa*
no	=>	*No /Maya*

Q: Were you in a vehicle or bus with more than 5 persons during the last 7 days?/Ma la raacday gaari ama bas in ka badan 5 qof toddobaadkii la soo dhaafay? (*)

Type: choice

A: one of the following:

yes	=>	*Yes /Haa*
no	=>	*No /Maya*

Q: Were you in a public gym in the past 7 days ?/Ma tagtay GYM dadweyne toddobaadkii la soo dhaafay?(*)

Type: choice

A: one of the following:

yes	=>	*Yes /Haa*
no	=>	*No /Maya*

Q: Did you go to a beauty parlor, massages, spa, hairdresser or nail studio in the past 7 days ?/Ma aadday saloon, massaj, SPA, timajare, ama ciddiyajare iwm toddobaadkii ugu danbeeyey? (*)

Type: choice

A: one of the following:

yes	=>	*Yes /Haa*
no	=>	*No /Maya*

Q: Did you go to a market in the past 7 days ?/Suuqa ma aadday toddobaadkii ugu tanbeeyey? (*)

Type: choice

A: one of the following:

yes	=>	*Yes /Haa*
no	=>	*No /Maya*

Q: Have you been using common plates or spoons when eating together with family members in the past 7 days ?/Ma la isticmaashay saxammo ama qaaddooyin qoyskaaga toddobaadkii ugu danbeeyey? (*)

Type: choice

A: one of the following:

yes	=>	*Yes /Haa*
no	=>	*No /Maya*

Q: Have you been using common plates or spoons when eating together with non-family members in the past 7 days ?/Ma la isticmaashay saxammo ama qaadooyin dad aan qoyskaaga ahayn toddobaadkii ugu danbeeyey? (*)

Type: choice

A: one of the following:

yes	=>	*Yes /Haa*
no	=>	*No /Maya*

Q: Did you travel in the past 7 days?/ma safartay toddobaadkii ugu danbeeyey? (*)

Type: choice

A: one of the following:

yes_other_provinces	=>	*Yes I traveled to other provinces/Haa waxaan u safray gobollo kale*
yes_outside_country	=>	*Yes I traveled outside the country/Haa waxaan u safray wadanka dibaddiisa*
no_travel	=>	*No travel/Ma safrin*

Q: During the last week, how worried or afraid were you about the health of your loved ones?/Toddobaadkii na dhaafay, sideed u walawalsaneyd ama u cabsaneysey dadkaad jeceshahay? Adigo ku cabbiraya 1= ma walwalsaneyn- ilaa 5= aad ayaan u walwalsanaa (*)

Type: choice_scale

A: 1 = not worried/ma walwalsaneyn to 5 = extremely worried/aad ayaan u walwalsanaa

1	=>	*1*
2	=>	*2*
3	=>	*3*
4	=>	*4*
5	=>	*5*

Q: On a scale of 1 to 10, can you indicate the extent to which people in your environment have practically adapted their behavior to the government recommendations?/Adigoo ku cabiraya 1 ilaa 10, ma qiyaasi kartaa ila heerka ay dadka deegaankaaga ku nooli u hirgeliyeen talooyinkii dawladda? (*)

Type: choice_scale

A: 1 = no adaptions,/mahirgaliyaan 10 = very strong adaptation/Aad ayay uhirgaliyaan

1	=>	*1*
2	=>	*2*
3	=>	*3*
4	=>	*4*
5	=>	*5*
6	=>	*6*

7	=>	*7*
8	=>	*8*
9	=>	*9*
10	=>	*10*

Questions related to your personal health/Su'aalo la xiriira caafimaadkaaga shaqsiga ah

Q: Have you been eating more healthy food such as fruits and vegetables since the coronavirus epidemic started?/Ma cunaysay cunto caafimaad leh, sida qudaarta ilaa iyo intii coronavirus uu bilowday? (*)

Type: choice

A: one of the following:

yes	=>	*Yes /Haa*
no	=>	*No /Maya*

Q: Have you been taking more vitamin tablets since the coronavirus epidemic started?/Ma liqdaa kiniin fitamiin ah? (*)

Type: choice

A: one of the following:

yes	=>	*Yes /Haa*
no	=>	*No /Maya*

Q: Did you have flu-like symptoms in the last 7 days (cough or sore throat, shortness of breath, headaches, body pains, fever)?/Ma ku qabteen astaamaha hargabku toddobaadkii ugu danbeeyey (qufac, dhuun-xanuun, neefsashada oo kugu adkaata, madax-xanuun, jir-xanuun, qandho/xummad)? (*)

Type: choice

A: one of the following:

yes	=>	*Yes /Haa*
no	=>	*No /Maya*
do_not_know	=>	*Do not know/Ma garanayo*

Q: If yes, which symptoms did you experience? (multiple options possible)/ Haday haa tahay imisa astaam ayaad isku aragtay(Jawaabo badan ayaad bixin kartaa) (*)

Type: choice_multiple

A: multiple answers possible:

fever	=>	*Fever/Qandho/Xummad*
headaches	=>	*Headaches/Madax-xanuun*
sore_throat	=>	*Sore throat/dhuun-xanuun*
loss_taste	=>	*Loss of taste/dhadhan la'aan*
loss_smell	=>	*Loss of smell/Ur la'aan*
stuffy_nose	=>	*Stuffy and/or runny nose/sanka xiran ama diif*
dry_cough	=>	*Dry cough/Qufac qalalan*
productive_cough	=>	*Productive cough/Qufac cantuuf leh*

shortness_breath	=>	*Shortness of breath/Neefsashada oo kugu adkaata*
muscle_pain	=>	*Muscle or body pains/Murqaha ama jir xanuun*
weakness	=>	*General weakness/ Tabardarro*
nausea	=>	*Nausea/Lalabo*
diarrhea	=>	*Diarrhea/shuban*

Visible if

Q:	A:
Did you have flu-like symptoms in the last 7 days (cough or sore throat, shortness of breath, headaches, body pains, fever)?/Ma ku qabteen astaamaha hargabku toddobaadkii ugu danbeeyey (qufac, dhuun-xanuun, neefsashada oo kugu adkaata, madax-xanuun, jir-xanuun, qandho/xummad)?	- value => yes

Q: For how many days did you have flu-like symptoms?/ Meeqa maalmood ayey ku hayeen astaamaha hargabku? (*)

Type: number

A: number (min: 1 / max: 50 / step: n/a)

Visible if

Q:	A:
Did you have flu-like symptoms in the last 7 days (cough or sore throat, shortness of breath, headaches, body pains, fever)?/Ma ku qabteen astaamaha hargabku toddobaadkii ugu danbeeyey (qufac, dhuun-xanuun, neefsashada oo kugu adkaata, madax-xanuun, jir-xanuun, qandho/xummad)?	- value => yes

Q: Are these symptoms still present?/Weli-se astaamaha hergebka ma ku hayaan? (*)

Type: choice

A: one of the following:

yes	=>	*Yes /Haa*
no	=>	*No /Maya*

Visible if

Q:	A:
Did you have flu-like symptoms in the last 7 days (cough or sore throat, shortness of breath, headaches, body pains, fever)?/Ma ku qabteen astaamaha hargabku toddobaadkii ugu danbeeyey (qufac, dhuun-xanuun, neefsashada oo kugu adkaata, madax-xanuun, jir-xanuun, qandho/xummad)?	- value => yes

Q: How many days ago did the symptoms disappear?/ Meeqa maalmood ka hor ayay astaamahaasi kaa tegeen?

Type: number

A: number (min: 1 / max: 50 / step: n/a)

Visible if

Q:	A:
Are these symptoms still present?/Weli-se astaamaha hergebka ma ku hayaan?	- value => No

Q: Have you been tested for COVID-19?/Ma lagaa baaray COVID-19? (*)

Type: choice

A: one of the following:

yes	=>	*Yes /Haa*
no	=>	*No /Maya*

Q: If yes, what was the result of the test? /Hadday haa tahay, maxuu ahaa natiijada ? (*)

Type: choice

A: one of the following:

positive	=>	*Positive/Bositive*
negative	=>	*Negative/Negative*
do_not_know	=>	*Do not know yet/ Ma garanayo*

Visible if

Q:	A:
Have you been tested for COVID-19?/Ma lagaa baaray COVID-19?	- value => yes

Q: Have any of your housemates had flu-like symptoms in the last 7 days?/Dadka kula nool ma lahaayeen astaamaha hargabka toddobaadkii la soo dhaafay? (*)

Type: choice

A: one of the following:

yes	=>	*Yes /Haa*
no	=>	*No /Maya*
do_not_know	=>	*Do not know/ Ma garanayo*

Q: Do you smoke?/Sigaarka ma cabtaa? (*)

Type: choice

A: one of the following:

yes	=>	*Yes /Haa*
no	=>	*No /Maya*

Q: Do you have an underlying disease (e.g. heart disease, asthma, diabetes, hypertension, cancer, HIV, tuberculosis, etc)?/Ma leedahay cudurro aasaasi ah (sida wadna xanuun, neef, sonkor, dhiig-kar, kansar, HIV, tiibisho/qaaxo, iwm.) (*)

Type: choice

A: one of the following:

yes	=>	*Yes /Haa*
not_to_my_knowledge	=>	*Not to my knowledge/Maya intaan ogahay*

Q: If you have an underlying disease did you experience difficulties to obtain your medication since the COVID outbreak started?/haddii aad cudur aasaasi ah qabtid, ma kugu adkaaday helitaanka dawooyinkaad u baahneyd ilaa iyo intii coronavirus uu dilaacay? (*)

Type: choice

A: one of the following:

Yes	=>	*Yes*
No	=>	*No*

Visible if

Q:	A:
Do you have an underlying disease (e.g. heart disease, asthma, diabetes, hypertension, cancer, HIV, tuberculosis, etc)?/Ma leedahay cudurro aasaasi ah (sida wadna xanuun, neef, sonkor, dhiig-kar, kansar, HIV, tiibisho/qaaxo, iwm.)	- value => yes

Q: I fully understand what this study is about, and I consent to participate. All the information I provide can be used by researchers to better understand coronavirus disease in my Country./Waxaan si buuxda u fahamsanahay waxa ay draasaddani ku saabsan tahay, waana oggolahay inaan ka qaybqaato. Dhammaan xogta aan bixinayo waxaa isticmaali kara cilmibaarayaasha si ay si fiican ugu fahmaan cudurka CORONAVIRUS ee ka jira gudaha dalkayga. (*)

Type: checkbox

A: checkbox

Appendix 2:

Model AIC during backward stepwise process

**Variables Removed**	**Degrees of freedom**	**Model AIC**
No variable removed		**27806**
Age	1	27807
Presence of underlying disease	1	27808
Residential setting	2	27810
Living alone in house	1	27811
Survey round	1	27812
Profession	4	27813
COVID-19 information from social media	1	27820
Educational level	3	27829
Presence of flu symptoms	1	27878
COVID-19 information from TV, radio, or government (Official source)	1	27880
Gender	1	27975
Healthcare student / worker	1	28126

Appendix 3:

COVID-19 preventive behaviours by gender

Characteristics	Survey 1		P-value	Survey 2		P-value
	Male (n=2490)	Female (n=1626)		Male (n=2768)	Female (n=1916)	
Wear face mask	1022 (41.0%)	1085 (66.7%)	< 0.001	1373 (49.6%)	1259 (65.7%)	< 0.001
Observe physical distancing	1475 (59.2%)	1155 (71.0%)	< 0.001	1555 (56.2%)	1215 (63.4%)	< 0.001
Wash hands regularly	1906 (76.5%)	1422 (87.5%)	< 0.001	1964 (71.0%)	1513 (79.0%)	< 0.001
Use hand gels regularly	1302 (52.3%)	1044 (64.2%)	< 0.001	1488 (53.8%)	1191 (62.2%)	< 0.001
Cover mouth when coughing	2110 (84.7%)	1489 (91.6%)	< 0.001	2229 (80.5%)	1636 (85.4%)	< 0.001
Avoid touching face	1665 (66.9%)	1150 (70.7%)	0.010	1682 (60.8%)	1301 (67.9%)	< 0.001
Stay home if symptoms	2007 (80.6%)	1451 (89.2%)	< 0.001	1939 (70.1%)	1471 (76.8%)	< 0.001
Mean adherence score (SD)	3.31 (1.52)	3.89 (1.33)	< 0.001	3.22 (1.63)	3.66 (1.49)	< 0.001
Been to bar/restaurant last 7 days	447 (18.0%)	77 (4.7%)	< 0.001	1319 (47.7%)	508 (26.5%)	< 0.001
Been to market last 7 days	1370 (55.0%)	778 (47.8%)	< 0.001	1814 (65.5%)	1124 (58.7%)	< 0.001
Travelled during past è days	174 (7.0%)	41 (2.5%)	< 0.001	539 (19.5%)	275 (14.4%)	< 0.001
Flu-like symptoms last 14 days	425 (17.1%)	240 (14.8%)	0.054	1046 (37.8%)	734 (38.3%)	0.742
